# Analysis of navigational risk indicators as a function of the ship's domain width for the selected offshore wind farm in the Baltic Sea

**DOI:** 10.1038/s41598-023-36114-3

**Published:** 2023-06-07

**Authors:** Grzegorz Rutkowski, Maria Kubacka

**Affiliations:** 1 Department of Navigation, Faculty of Navigation, Gdynia Maritime University, 81-225, Gdynia, Poland; 2 Department of Operational Oceanography, Maritime Institute, Gdynia Maritime University, 80-830, Gdansk, Poland

**Keywords:** Ecology, Environmental social sciences, Hydrology, Energy science and technology, Engineering

## Abstract

This study concerns the analysis of navigational risk indicators as a function of the ship's domain width estimated for nine selected representative ships sailing under various hydrometeorological conditions (average and deteriorated ones) observed within the Offshore Wind Farm to be constructed within the Polish offshore zone on the Baltic Sea. For this purpose, the authors compare three types of domain parameters according to the guidelines by the PIANC, Coldwell and Rutkowski (3D). The study enabled selection of a group of ships which can be considered safe and can optionally be allowed to navigate and/or fish in the immediate vicinity and within the offshore wind farm. The analyses required the use of hydrometeorological data, mathematical models and operating data obtained with the use of maritime navigation and manoeuvring simulators.

## Introduction

The Baltic Sea has shallow waters, high average wind speeds, low wave heights and weak tides. Such conditions result in low levelized cost of energy (LCOE) values for offshore wind energy production and make the Baltic Sea a prospective area for the development of offshore wind farms (OWFs). To date, offshore wind turbines have been installed in Denmark, Germany, Sweden and Finland, yet there is no wind farm within the Polish Exclusive Economic Zone (EEZ). Poland is the last EU country at the pre-development and consenting stages; however, many pre-investment studies and survey campaigns have been carried out for several of such investments^1–4^. Currently, eight projects have secured contracts-for-difference (CfDs) granted by the Polish Energy Regulatory Office (ERO) as part of an administrative procedure introduced under the Offshore Wind Act. As declared by the investors, the most advanced projects should be commissioned between 2026 and 2027^2^.

The Baltic Sea is one of the busiest seas in the world, with sea transport accounting for 15% of global sea freight^5^. According to the Statistics Poland^6^, cargo turnover, passenger traffic and the number of ships calling at Polish seaports have increased over the past years. Apart from transport and tourism, human activity at sea is also related to the petroleum and seafood industries. When analysing the above, it becomes clear that offshore windmills installed will become navigational obstructions affecting the safety of navigation^7–16^. Therefore, it is necessary to establish safety zones for representative ships sailing in the vicinity of OWFs, assess their safety of navigation while manoeuvring within the OWF areas and estimate their so-called navigational risk indicators.

In the Baltic Sea region, different regulatory regimes are applied for vessel traffic through wind farms. For example, in Belgium and Germany, wind farms are considered maritime exclusion zones to prevent accidents or damage to turbines, whereas, in the UK and Denmark, wind farms are open for shipping, and both commercial and recreational use. In Denmark, for example, wind farms are open to transit for ships of up to 24 m in length. Such operations may only take place during the day with the VHF and AIS system being operational and activated. Seabed-disturbing activities and third-party diving activities are forbidden within offshore wind farms. Safety zones of 50 m are established around the turbines and the 500-m safety zones around offshore transformer stations remain in place. In the case of new offshore wind farms, the establishment of a corridor is being considered to make it possible for vessels of up to 45 m to travel through them^17^.

The British requirements for safe navigation guidelines for offshore renewable energy installations (UK Maritime and Coastguard Agency, 2016) provide the following recommendations for estimating the safe distance of a turbine from the shipping route:

1. If the distance between the turbine boundary and the shipping route is less than < 0.5 nm (< 926 m), it is deemed intolerable;
2. If the distance is between 0.5 and 3.5 nm (926–6482 m), it is deemed tolerable provided that the risk being reduced to as low as reasonably practicable (ALARP)—additional risk assessment and proposed mitigation measures required;
3. If the distance is more than > 3.5 nm (> 6482 m), it is deemed broadly acceptable.

Currently, the Polish maritime administration bodies, acting under Art. 24 in connection with Art. 47 of the Act of 21 March 1991 on maritime areas of the Republic of Poland, are considering the introduction of safety zones around structures and devices constituting elements of OWFs situated within the maritime areas of the Republic of Poland. At the moment of writing this paper, the above-mentioned legal regulations have not been developed and/or published on the official websites of the Polish maritime administration bodies.

General guidelines regarding the risks of navigation and safety zones in the vicinity of OWFs were presented by the World Association for Waterborne Transport Infrastructure PIANC^18^, according to which the level of navigational risk from the OWF impacts depends on the distance between a Traffic Separation System (TSS) shipping route and the first row of wind turbines. According to the PIANC guidelines, the level of unacceptable risk will be estimated for ships to which the SOLAS Convention^19^ applies, and which are manoeuvred at a distance of less than 0.25 NM (463 m) and/or 500 m from the designated high-density shipping routes.

According to the PIANC, ships navigating within the TSS area situated at a distance of more than 5 NM (≈ 9260 m) from the OWF can be considered to be safe in restricted sea areas. As per the PIANC guidelines^18^, the minimum distance that guarantees the safety of navigation refers to the COLREG regulations^20^ and is determined based on the resolutions of IMO^21–23^, MSC.137 (76)^22^ and MSC/Circ.1053^21^, which address ship manoeuvrability and, in particular, the parameters of the turning circle manoeuvre and the emergency stopping (decelerating) distance. According to the PIANC guidelines, the minimum safe distance from a navigational obstacle that defines the ship’s domain should be determined using the following formulae:

1 $$d_{NP} = SD_{WP} = 6 \cdot LOA + 500 m$$

2 $$d_{NS} = SD_{WS} = d_{NP} + 0.3 Mm \approx 6 \cdot LOA + 1056 m$$

where ${d}_{NP}$ = the minimum distance from a navigational obstacle situated on the ship’s port side identified with the ship’s domain on the port side (*SD*_*WP*_); expressed in meters, [m]; ${d}_{NS}$ = The minimum distance from a navigational obstacle situated on the ship’s starboard side identified with the ship’s domain on the starboard side (*SD*_*WP*_); expressed in meters, [m]; LOA = ship’s length overall expressed in meters, [m].

**Figure 1:**
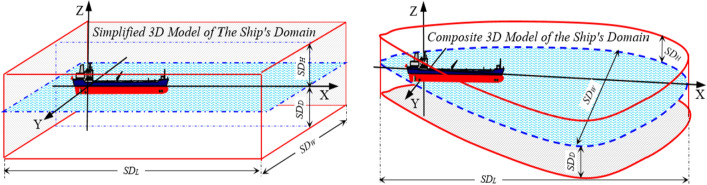
Simplified and composite approaches for the three-dimensional (3D) model of the ship's domain with its length (*SD*_*L*_), width (*SD*_*W*_), depth (*SD*_*D*_) and height (*SD*_*H*_). A model based on G. Rutkowski’s own scientific research.

The term ‘ship’s domain’^24^ has been widely analyzed in the existing literature concerning the safety of shipping^25–28^ and assessment of the navigational collision risk^26,29,30^, and is defined as the area around a vessel which is indispensable for maintaining the safety of navigation. Therefore, the navigational risk increases when any navigational obstruction appears within the ship’s domain. Most of the proposed ship’s domain models are two-dimensional (2D)^28,31^ rather than spatial (3D)^27,32^. This paper compares three domain models according to the guidelines by the PIANC, Coldwell, and Rutkowski (3D). The ship’s domain by Rutkowski (3D), which was developed based on the author’s own research, is presented in Fig. 1.

**Figure 2:**
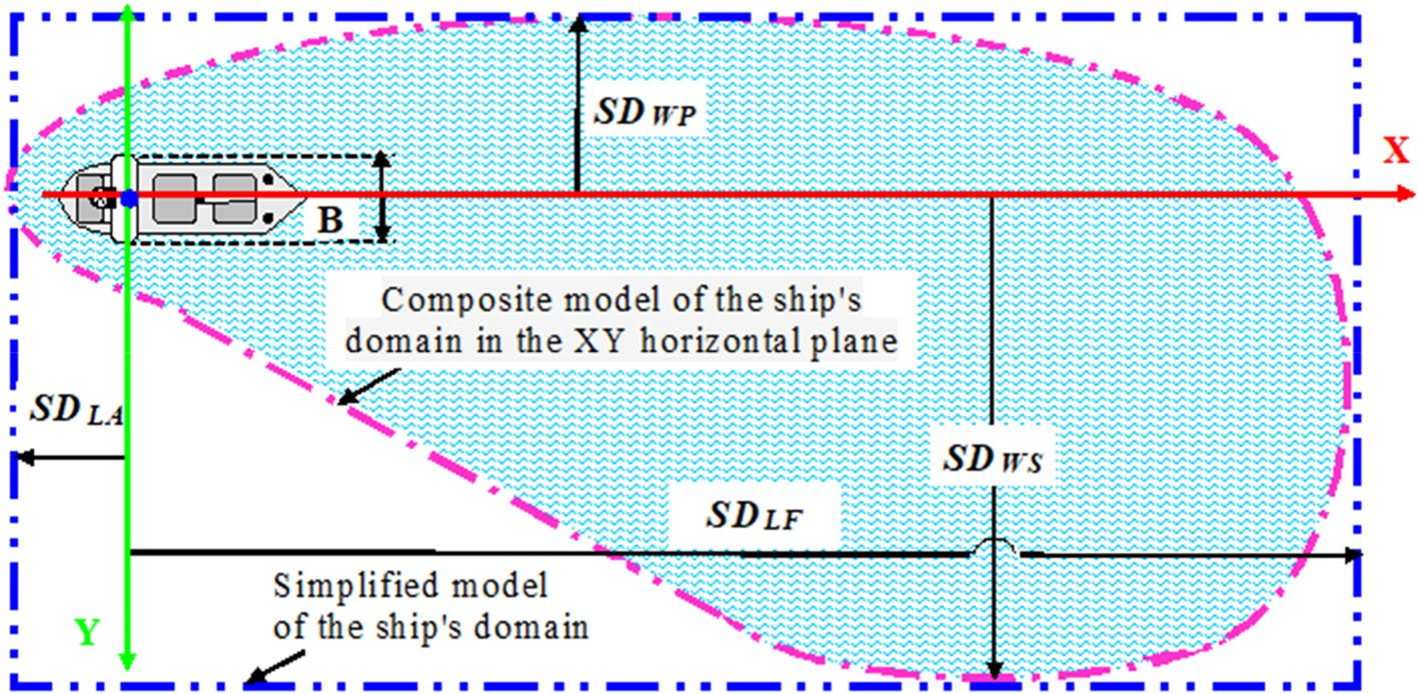
Simplified and composite approaches for the 3D model of the ship's domain in the XY horizontal plane with its length forward (*SD*_*LF*_), length aft (*SD*_*LA*_), width to port (*SD*_*WP*_) and width to starboard (*SD*_*WS*_). A model based on G. Rutkowski’s own scientific research.

Figure 2 illustrates the simplified and composite approaches for the 3D model of the ship’s domain in the XY horizontal plane with its length forward (SD_LF_), length aft (SD_LA_), width to port (SD_WP_), and width to starboard (SD_WS_). However, due to the limited nature of our work, this paper focuses only on the analysis of two of the six parameters of the 3D model of the ship’s domain by Rutkowski^12^, and, in particular, the ship's domain width in the horizontal plane on the port side (SD_WP_) and the starboard side (SD_WS_) of the ship.

## Research objectives

This study focused on the following research objectives:

- determining navigational risk numeric indicators *R*_*NWP*_ and* R*_*NWS*_ with respect to keeping the required width for the safety of the vessel traffic lane on the ship’s port and starboard sides as estimated for a group of representative ship types which may navigate within OWF areas;
- selecting a group of ships from among the representative ship types, which may pose a particular hazard to the OWF operation, and a group of ships which can be considered safe and can optionally be allowed to navigate and/or fish in the immediate vicinity and within OWFs;
- comparing the domain parameters for the selected representative ship types compiled according to the guidelines by the PIANC, Coldwell and Rutkowski (3D).

## Materials and methods

### The navigational risk with respect to keeping the required ship’s domain width

According to the definition of navigational risk (*R*_*N*_)^12^, a risk coming from factors *A*_i_ (objects) and equal to 0 denotes full safety of navigation with respect to these factors (objects). Analogously, the higher the risk (parameter *R*_*N*_ approximating 1), the lower the level of the safety of navigation (*S*_*N*_) → (*R*_*N*_ + *S*_*N*_ = *1; S*_*N*_ = *1 − R*_*N*_). Therefore, the navigational risk indicator reaching *R*_*N*_ = 1 denotes the occurrence of such conditions and/or circumstances which are going to prevent safe navigation and may entail a 100% probability of a collision.

*R*_*N*_ will be analysed in this paper based on the definition of the ship’s domain (*SD*)^12^ and the definition of *R*_*N*_^12,13^, the values of which can be determined with reference to the vertical plane OX and the horizontal plane OY^33^. The analysis will further focus, in particular, on the components of *R*_*N*_ defined with reference to the OY plane and in relation to objects situated on the ship’s port side (*R*_*NWP*_), and the ship’s starboard side (*R*_*NWS*_), which can be presented with the use of the following formulae:

3 $$R_{NWP} = \left\{ {\begin{array}{*{20}c} 0 & {{\text{when}}} & {d_{NP} > SD_{WP} } \\ {\frac{{SD_{WP} - d_{NP} }}{{SD_{WP} - 0.5 \cdot B}}} & {{\text{when}}} & {\frac{B}{2} < d_{NP} \le SD_{WP} } \\ 1 & {{\text{when}}} & {d_{NP} \le \frac{B}{2}} \\ \end{array} } \right.$$

where *R*_*NWP*_ is a dimensionless value defining a component of *R*_*N*_ with respect to keeping the required safe width of the ship's passage route (distance d_NP_ from the nearest navigational danger situated on the OY axis) on the ship’s port side related to the possibility of the ship colliding with a navigational obstacle situated on the ship’s port side; *SD*_*WP*_ (*Ship’s Domain Width Port Side*) is the ship’s domain width as measured on the ship’s port side. It is expressed in meters measured along the OY axis perpendicular to the ship’s heading (true course line *TC*) on the ship’s port side; *d*_*NP*_ is the distance from the nearest hazard (navigational danger) measured in meters perpendicular to the ship’s heading (true course line *TC*) on the ship’s port side; *B* is the ship’s width in meters as per the ship’s particulars, the pilot card or the AIS.

4 $$R_{NWS} = \left\{ {\begin{array}{*{20}c} 0 & {{\text{when}}} & {d_{NS} > SD_{WS} } \\ {\frac{{SD_{WS} - d_{NS} }}{{SD_{WS} - 0.5 \cdot B}}} & {{\text{when}}} & {\frac{B}{2} < d_{NS} \le SD_{WS} } \\ 1 & {{\text{when}}} & {d_{NS} \le \frac{B}{2}} \\ \end{array} } \right.$$

where *R*_*NWS*_ is a dimensionless value defining a component of *R*_*N*_ with respect to keeping the required safe width (distance *d*_*NS*_ from the nearest navigational hazard situated on the OY axis) on the ship’s starboard side (index *WS* = *Width Starboard Side*). This parameter describes the navigational risk (estimated as ranging from 0 to 1) related to the possibility of the ship colliding with a navigational obstacle on the ship’s starboard side (*Adequate Required Safe Distance from the Nearest Danger on Ship’s Starboard Side*); *SD*_*WS*_ (*Ship’s Domain Width Starboard Side*) is the ship’s domain width as measured on the ship’s starboard side. It is expressed in meters measured along the OY axis perpendicular to the ship’s heading (true course line *TC*) on the ship’s starboard side, [m]; *d*_*NS*_ is the distance from the nearest hazard measured in meters perpendicular to the ship’s course line on the ship’s starboard side, [m].

According to the ship’s domain definition^12^, every ship will be safe (in navigational meaning) as long as she is the exclusive object capable of generating hazards within her domain.

With reference to the horizontal plane OY distinction between R_NWP_ and R_NWS_ of the navigational risk R_N_, which can be referred to as the horizontal components of the navigational risk related to keeping a safe distance from the nearest danger adequately on the port and starboard sides of the ship, or, in short, the risk of keeping a safe distance from the port and starboard sides, can be depicted by means of formulas (3) and (4). According to the patterns presented above, (*R*_*NWP*_ formula 3) with the (*d*_*NP*_ > *SD*_*WP*_) condition and (*R*_*NWS*_ formula 4) with the (*d*_*NS*_ > *SD*_*WS*_) condition guarantee safe navigation of the ship in relation to the objects detected on the ship’s starboard side and port side respectively. When analysing formulas 3 and 4, one can also notice that the value of navigational risk R_NW_ will be limited to a range between zero and one (*R*_*NW*_* ϵ*^7^) only if the distance from the nearest danger on the port side (d_NP_) or starboard side (d_NS_) is either less or equal to the ship’s domain width calculated respectively for the ship’s port side (*SD*_*WP*_) and/or starboard side (*SD*_*WS*_). In all probability, assumption ${d}_{N}\le \frac{B}{2}$ indicates a navigational accident or collision with some objects (obstructions) detected respectively on the ship’s port side (formula 3$: {d}_{NP}\le \frac{B}{2}$) and/or starboard side (formula 4: ${d}_{NS}\le \frac{B}{2}$) and/or an unquestionable (100%) risk of collision with those objects.

**Figure 3:**
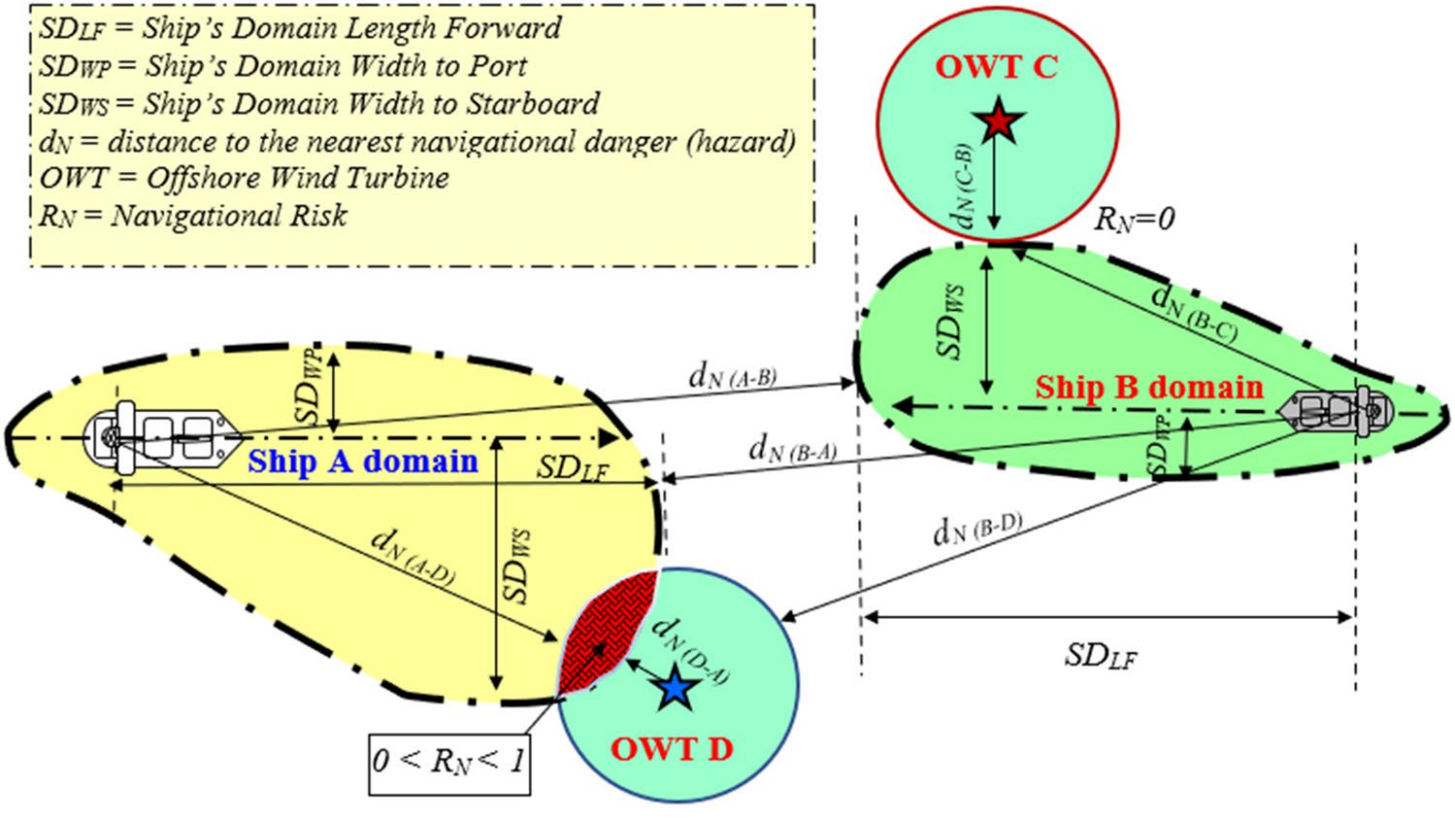
A graphical display of the navigational risk indicators (*R*_*N*_) as a function of the ship’s domain parameters (*SD*_*WP*_, *SD*_*WS*_) and the distance from the nearest navigational obstruction (*d*_*N*_).

A graphical display of *R*_*N*_ as a function of the ship’s domain parameters (*SD*_*WP*_*, SD*_*WS*_) and distance from the nearest navigational hazard (*d*_*N*_) is presented in Fig. 3. The analysed *R*_*N*_ factors in the horizontal plane OY in relation to the objects situated on the ship’s port and starboard sides obtained for different ship types navigating within the OWF sea area are presented below.

### Representative ship types

For the purposes of the paper, our analysis covered nine representative ship types (Table 1) with mathematical models and operating (manoeuvring) data obtained with the use of the maritime navigation and manoeuvring simulators provided by the Faculty of Navigation of the Gdynia Maritime University:

- Polaris Ships Bridge simulator, Version 8.0.0 Build 384 with a DP-K-Pos dynamic positioning simulator by Kongsberg Digital A.S. (Ship’s Models as per Kongsberg Digital Doc no.: SO-0609-E7/ 22.04.2017, Polaris Ship’s Bridge Simulator Technical Manual Section 2—Technical data, v.7.6.0);
- K-Sim Navigation (*full-mission bridge*) by Kongsberg Digital A.S. (Ship’s Models as per Kongsberg Digital Doc no.: SM-0521-J / 26.08.2016, Appendix B—Hydrodynamic Models NO.:SM-0521-K / 26.08.2016 K-Sim Ship's Bridge Simulator), and
- NaviTrainer 5000 Professional (Ship’s Models as per Wärtsilä Navi-Trainer Professional 6, Technical Description and Installation Manual Version 6.0, Date of issue: December 2022) combined with an electronic chart system ECDIS NaviSailor 4000 by Transas, which is part of the Wärtsilä Group.

**Table 1:** The representative ship types.

Ship symbol	Ship type	DWT [t]	Engine power [kW]	Length overall LOA [m]	Width B [m]	Draft (Tmax) [m]	Overall height (H_c_) [m]	Air draft (H_N_) [m]	Block coefficient C_B_	Full speed ahead (FSAH)
A	VLCC (very large crude carrier)	159,584	15,500	261.3	48.3	15	77.3	62.3	0.85	15.0 kn, ca. 7.7 m s^−1^
B	Bulk carrier	33,089	8827	182.9	22.6	10.7	42.7	32	0.83	14.0 kn, ca. 7.2 m s^−1^
C	Lo-Ro (lift-on/lift-off, roll-on/roll-off) ship	19,512	9540	173.5	23	8.1	51	43	0.68	18.9 kn, ca. 9.7 m s^−1^
D	Fisher ship	1676	840	65.6	10.3	5.4	32.3	27	0.64	12.6 kn, ca. 6.5 m s^−1^
E	High speed water jet rescue ship	12	2 × 331	12.2	4.2	0.7	3.7	3	0.56	38 kn, ca. 19.5 m s^−1^
F	Fishing boat	286	440	24.4	7.2	3.7 (TD 2.6 m; TR 3.7 m)	18.5	14.8	0.7	11 kn, ca. 5.7 m s^−1^
G	Container ship	93,130	54,847	279	40.4	14.02	71.5	57.5	0.598	27.1 kn, ca. 13.9 m s^−1^
H	LNG carrier	108,957	26,800	297.5	45.7	10.75	73.1	62.3	0.62	20.3 kn, ca. 10.4 m s^−1^
I	Z-drive prevention response tug	300	2 × 3800	45	12.5	4.9	21	16.1	0.614	FSAH 15.0 kn, ca. 7.2 m s^−1^; HAH 5.7 kn, ca. 2.9 m s^−1^

### Hydrometeorological data: average and deteriorated conditions

The parameters of spatial domain models were estimated for representative ship types navigating under average hydrometeorological conditions adequate for the analysed navigable sea area and under deteriorated conditions. The weather parameters and information about the hydrological conditions prevailing in the southern Baltic Sea area as necessary for safe navigation across this basin are presented in Table 2. The data come from the publication entitled the Sailing Directions^34^. The information concerns the waters of the Baltic Sea along the Polish coast and may refer to the Project area. The weather parameters and hydrological conditions presented cover average values obtained during many years of research.

**Table 2:** The average and deteriorated conditions. *Source*:^34^.

Parameter	Average conditions	Deteriorated conditions
Visibility	At least 5 NM	Reduced to 2 NM
Wave height	*hf* ≈ 1.0 m	*hf* ≈ 3.0 m
Wind	3–4°B	5–6°B
Permanent surface current velocity	*Vp* ≤ 0.2 kn	Vp ≤ 0.4 kn
Current direction (Kp)	In line with the direction of the vessel traffic stream within the TSS	Perpendicular to the direction of the vessel traffic stream within the TSS
Water level vertical oscillations referred to chart datum (Chart Datum = MSL)	± 0.3 m	Not more than ± 0.60 m
Water density (ρ)	1.0066 g cm^−3^	1.0066 g cm^−3^
Ship drift angle (a)	Not more than ± 1°	Not more than ± 2°
Maximum yawing (∆)	Up to ± 1°	Up to ± 2°
Roll angle (a)	Up to ± 1°	Up to ± 3°

### Determining domain parameters for representative ship types

For the purposes of the paper, we referred to three spatial models of the ship’s domains: the PIANC guidelines^18^ (formulas 1 and 2), the 2D domain by Coldwell^31^:

5 $$SD_{WP} = 1.75 \cdot LOA$$

6 $$SD_{WS} = 3.25 \cdot LOA$$

and the 3D domain by Rutkowski described with reference to the XYZ coordinate system by G. Rutkowski in 2000–2021^12,13,24,32,33,35,36^:

7 $$SD_{{{\text{WP}}}} = p \cdot \left( {\frac{{B_{C} }}{2} + {\Delta B}} \right) + {30}{\text{.87}} \cdot t_{r} \cdot SOG \cdot {\text{sin}}\left( {{\text{COG}} -{^\circ{TC}}} \right) + r_{W} \cdot \left[ {s_{W} \cdot TR_{neg} + {30}{\text{.87}} \cdot t_{m} \cdot {\text{ Drift}} \cdot \sin \left( {Set -{^\circ{TC}}} \right)} \right]$$

8 $$SD_{{{\text{WS}}}} = p \cdot \left( {\frac{{B_{C} }}{2} + {\Delta B}} \right) + {30}{\text{.87}} \cdot t_{r} \cdot SOG \cdot {\text{sin}}\left( {{\text{COG}} -{^\circ{TC}}} \right) + r_{W} \cdot \left[ {s_{W} \cdot TR_{{{\text{max}}}} + {30}{\text{.87}} \cdot t_{m} \cdot {\text{ Drift}} \cdot \sin \left( {Set -{^\circ{TC}}} \right)} \right]$$

where *SOG* = ship’s speed over ground in knots obtained from doppler log or fix ship’s positioning system such GNSS/GPS, (*SOG* = *V*_*d*_) where $\overrightarrow{{V}_{d}}=[COG, SOG]$, the value expressed in knots, [kn], *COG* = ship’s course over ground ($\overrightarrow{{V}_{d}}=[COG, SOG]$) expressed in degrees of angles, [°], *B* = ship’s width (beam) in meters based on the ship’s particulars, [m], *∆B* = a factor showing an increase in width (beam) of the ship’s domain. The increase amounts to error *M*_*OY*_ of the total ellipse errors *δ*_*y*_*(B*_*i*_*)* for all factors *B*_*i*_ that affect *SD*_*WS*_, estimated with the probability level of *p* = 95% (C = 2.44); in this paper, the following is assumed: *ΔB* = 10 m, *B*_*C*_ = Seeming width of the ship’s trace calculated horizontally in meters [m], with wind leeway angle *α* [°], current deviation (drift angle) β [°], and ship’s yawing Δ[°]:

9 $${\text{B}}_{{\text{C}}} = {\text{L}} \cdot {\text{sin}}\left( {{\upalpha } + {\upbeta } + {\Delta }} \right) + {\text{B}} \cdot {\text{cos}}\left( {{\upalpha } + {\upbeta } + {\Delta }} \right)$$

*TR*_*max*_ = the ship’s transfer maximum values measured in meters as the maximum movement of the ship to the port or starboard side (transverse horizontally to ship’s initial course line), observed after changing the course ∆*TC* ≥ 180° or after the ship’s stopping manoeuvre is completed, [m],

*TR*_*neg*_ = the ship’s ‘negative’ transfer (maximum value) measured in meters, observed after on the side opposite to the general direction during the ship’s turning and/or stopping manoeuvre, also known in maritime terminology as the ‘kick’ distance on turning circle diagrams. *TR*_*neg*_ is specified for merchant ships as a value between 1.0 and 1.5 of the ship’s breadth B (for turning circulation) or around 1.5 of the ship’s length L (for a Crash Stop (Full Ahead-Full Astern) emergency manoeuvre), [m]. *t*_*m*_ = the period of time needed to stop the ship or change the direction of the ship’s movement by ∆*TC* ≥ 090° expressed in minutes based on the Pilot Card, Wheel House Poster or Turning Circle Diagrams, [min], *t*_*r*_ = the period of time needed for the appropriate reaction, that is the right assessment of the navigational situation and giving a manoeuvre order. In practice, *t*_*r*_ ≈ 0.5 min up to 3.0 min depending on the seafarer competence and his professional experience, [min],

*Drift* = the total current speed value in knots (*Drift* = *V*_*z*_) where $\overrightarrow{{V}_{z}}=[Set, Drift]$, and total current = water flow = sea current + tide stream, [kn],

*Set* = the total current ($\overrightarrow{{V}_{z}}=[Set, Drift]$) direction in degrees,

*p* = a factor (numeric coefficient) depending on the harmfulness of the cargo carried on board the ship. This factor (1 ≤ *p* ≤ 2) increases the safety margin of navigational reserve in case of an abnormal situation, which can result either in a catastrophe (disaster) or contamination of the environment. In this paper, we recommend using the following values for factor *p*: for ships in ballast condition without dangerous cargo or harmless charge, neutral for people and the environment: *p* = 1; for ships carrying a load of high harm to people and the environment, e.g. flammable substances, oil, natural gas.: *p* = 1.5; for ships with a very harmful load for people and the environment, e.g. radioactive substances, corrosive chemicals, explosive substances: *p* = 2.0,*r*_*W*_ = a numeric coefficient (factor) correcting the width (*r*_*W*_) of the ship’s domain (0 ≤ *r*_*W*_ ≤ 2), depending on her situation (privilege) according to the COLREG Rules. In this paper, we recommend the following values for factor *r*_*W*_: for a ship aground or at anchor: *r*_*W*_ = 0; for ships restricted by their draught: *r*_*W*_ = 1; for privileged ships such as vessels with restricted ability to manoeuvre (except the vessels engaged in mine clearance and vessels engaged in fishing: *r*_*W*_ = 1.5; for sailing ships and ships that are not under command: *r*_*W*_ = 2, *s*_*W*_ = a numeric coefficient (factor) correcting the ship’s transfer (*TR*) parameter on turning circle in case of unexpected meteorological conditions other than those previously observed during sea trials and recorded in the Pilot Card and Wheel House Posters (currently excluded).

The parameters were estimated for the turning cycle manoeuvre with the ship at full sea speed ahead (FSAH) with the rudder angle of 35° starboard and emergency stop manoeuvres by reversing the engine to full astern (FSAH-FAS and HAH-FAS). The results are presented in the further part of this paper.

## Results and discussion

It has been assumed in this paper that the actual distances between individual offshore installations within the OWF area range from *d*_*min1*_ = 700 m (in the case of substations) and from *d*_*min2*_ = 1000 m to *d*_*min3*_ = 2000 m in the case of measuring distances between individual offshore wind turbines, however our analysis has been extended to address seven different distances: 300 m, 500 m, 600 m, 700 m, 800 m, 1000 m and 2000 m. When analysing emergencies, when ships are allowed to enter the OWF area, it was assumed that they sail at an optimal (maximum) distance from any navigational hazards detected nearby and situated respectively ahead of their bows and on their port and starboard sides. Here, an assumption can be made that, in the vicinity of substations, the minimum distance from the nearest hazard will be a value defined as half of the distance between individual offshore installations, i.e. *d*_*N1*_ = *0.5∙d*_*min1*_ = 350 m, and for the location of wind turbines within the OWF area, this will be the distance ranging from *d*_*N2*_ = *0.5∙d*_*min2*_ = 500 m to *d*_*N3*_ = *0.5∙d*_*min3*_ = 1000 m.

Table 3 presents the domain parameters for the nine ship types (Table 1) compiled as per the PIANC guidelines^18^, 2D domain by Coldwell and the method by Rutkowski^13^ using the manoeuvring characteristics obtained by the manoeuvring simulator of the Gdynia Maritime University calculated for average and deteriorated hydrometeorological conditions.

**Table 3:** The domain parameters for the following ship types: A (very large crude carrier), B (bulk carrier), C (Lo-Ro ship), D (fisher ship), E (high speed water jet rescue ship), F (fishing boat), G (container ship with DWT of 93,100), H (LNG carrier) and I (Z-drive prevention response tug), compiled as per the PIANC guidelines, Coldwell’s ship’s domain 2D model and the method by Rutkowski^13^ using the manoeuvring characteristics obtained by the manoeuvring simulator of the Gdynia Maritime University.

Ship type	PIANC guidelines		T.G. Coldwell’s ship’s domain 2D model		Rutkowski’s [after^13^] ship’s domain 3D model									
					Average condition						Deteriorated conditions			
					Turning circle manoeuvre at FSAH / rudder 35° STRB		FSAH-FAS manoeuvre as per Wheelhouse Poster		HAH-FAS manoeuvre as per Wheelhouse Poster		Turning circle manoeuvre at FSAH / rudder 35° STRB		FSAH-FAS manoeuvre as per Wheelhouse Poster	
	SD_WP_ [m]	SD_WS_ [m]	SD_WP_ [m]	SD_WS_ [m]	SD_WP_ [m]	SD_WS_ [m]	SD_WP_ [m]	SD_WS_ [m]	SD_WP_ [m]	SD_WS_ [m]	SD_WP_ [m]	SD_WS_ [m]	SD_WP_ [m]	SD_WS_ [m]
Ship A	2068	2624	457	849	153	970	472	603	392	523	221	1038	541	672
Ship B	1597	2153	320	594	73	725	314	405	274	366	138	790	379	470
Ship C	1541	2097	304	564	74	707	300	387	260	347	139	771	457	544
Ship D	894	1450	115	213	47	272	130	162	98	131	110	335	193	225
Ship E	573	1129	21	40	34	175	46	52	18	24	46	188	52	58
Ship F	646	1202	43	79	40	85	66	78	37	49	59	104	85	97
Ship G	2174	2730	488	907	111	1069	469	608	419	558	140	1099	535	675
Ship H	2285	2841	521	967	148	1136	526	675	446	595	218	1205	595	744
Ship I	770	1326	79	146	51	110	57	77	57	77	76	135	58	78

Data as of May 2022.

The following parameters and coefficients are used in this paper: n = 1.2 (rocky sea bottom); m = 1.0; k = 0.66; sL = 1.0; sW = 1.0; rL = 1.0; rW = 1.0; ΔL = 25 m; ΔB = 25 m; tr = 0.5 min; *p* = 1.0 (non-hazardous cargo); water density ρ = 1.0066 g cm^−3^; OHC_R_ = 3 m.

Table 4 presents sample navigational risk indicators ${{R}_{NWP}(SD}_{WP})$ and ${{R}_{NWS}(SD}_{WS})$ with respect to keeping the required distance from navigational hazards detected on the ship’s port side and starboard side respectively. These indicators were estimated for nine representative ship types (Table 1) as a function of the width of their domains calculated for average hydrometeorological conditions (Table 2). The navigational risk numeric factors ${R}_{NWP}$ and ${R}_{NWS}$ were estimated using the domain parameters ${SD}_{WP}$ and ${SD}_{WS}$ compiled in the tables (Table 3). In Table 4, the numeric indicators of *R*_*N*_ ranging from 0 to 33% (${0\le R}_{N}\le 0.33$) are assumed to be acceptable and are marked in shades of green colour. They denote a navigational situation for which the values of the estimated *R*_*N*_ factors are considered to be safe, making it possible to execute a voyage. The *R*_*N*_ numeric indicators ranging from 66 to 100% (${0.66\le R}_{N}\le 1$) are considered to be dangerous or highly risky, and are marked in shades of red colour. The *R*_*N*_ indicators representing middle values (${0.33<R}_{N}<0.66$) require additional intervention from the person conning the ship, and are marked in shades of yellow colour.

**Table 4:**
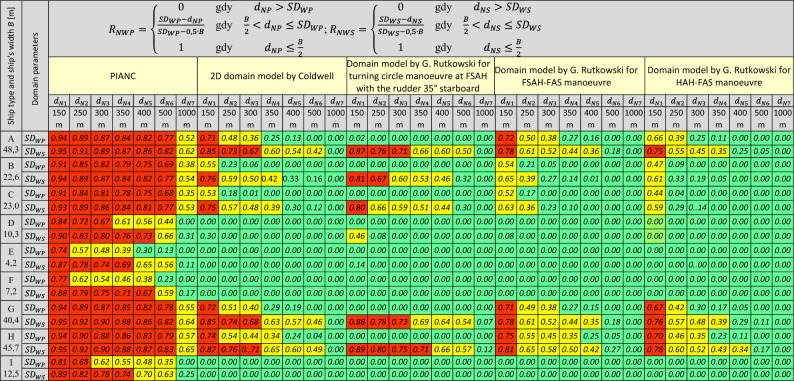
Presentation of the navigational risk numeric indicators ${{R}_{NWP}(SD}_{WP})$ and ${{R}_{NWS}(SD}_{WS})$ with respect to keeping the required distance from hazards on the port side and on the starboard side as estimated for the analysed area under average hydrometeorological conditions. *Source*: Prepared by G. Rutkowski based on^13^. May 2022.

Remarks: Red means a dangerous situation, green means a safe situation, and yellow means a doubtful situation that requires additional user intervention (correcting and/or mitigating actions).

According to the analysis of the indicators ${{R}_{NWP}(SD}_{WP})$ and ${{R}_{NWS}(SD}_{WS})$ (Table 4), depending on the method applied (in this case, the PIANC guidelines, the 2D domain by Coldwell and the 3D domain by Rutkowski estimated for the turning circle manoeuvres at FSAH with the rudder angle of 35º starboard, and emergency stop manoeuvres by reversing the engine to full astern FSAH-FAS and HAH-FAS), the *R*_*N*_ indicators sometimes assume radically different values. In addition, the PIANC method seems to be the most restrictive one (red fields in Table 4). However, according to the PIANC method, the values of the estimated ${{R}_{NW}(SD}_{W})$ depend on the overall dimensions of the analysed representative vessels to a small extent only, and, moreover, this method fails to take account of their actual manoeuvring parameters. Hence, it is doubtful whether this method should be used for practical estimation of the navigational risk factors for small surface vessels type D, E, F and I, for which, according to the PIANC method, for the distance from the nearest danger ${d}_{N1}=350 m$ on the ship’s port and starboard sides, the estimated navigational risk indicators range from 39% for ship E to 61% for ship D, 46% for ship F and 55% for ship I, taking into account the risk factors estimated for the port side: ${{R}_{NWP}(SD}_{WP})\in \left(0.39;0.46;0.55; 0.61\right),$ and from 69% for ship E to 76% for ship D, 71% for ship F and 74% for ship I, taking into account the risk factors estimated for the starboard side: ${{R}_{NWS}(SD}_{WS})\in (0.69;0.71;0.74;0.76)$.

As regards the 2D domain method by Coldwell and the 3D domain method by Rutkowski, navigating ship types D, E, F and I proves to be completely safe, taking into account the presence of navigational hazards situated on the ship’s port side and starboard side respectively. Moreover, the parameters of the 2D domain by Coldwell are very similar to those of the 3D domain by Rutkowski as estimated for the emergency turning circle manoeuvre performed at full speed ahead FSAH with the rudder angle of 35º starboard. Additionally, the 2D domain by Coldwell is an empirical domain estimated in the XY horizontal plane only and it does not take account of the navigational risk generated by above-water and underwater navigational obstacles. In addition, the 3D domain by Rutkowski allows for choosing the right anti-collision manoeuvre, which is performed by way of changing the course (turning circle manoeuvre) and/or changing the ship’s speed ahead (FSAH or HAH). For example, an analysis of ${{R}_{NWP}(SD}_{WP})$ and ${{R}_{NWS}(SD}_{WS})$ conducted for ship A (a VLCC), assuming that the distance from the nearest danger on the ship’s port and starboard sides is ${d}_{N2}=500m$, proves that performing a turning circle manoeuvre to starboard at full speed ahead FSAH will generate a navigational risk on the ship’s starboard side of 42% = ${{R}_{NWS}(SD}_{WS})=$ 0.42. In case of performing an emergency stop manoeuvre by reversing the engine to full astern FSAH-FAS, the navigational risk factor generated on the ship’s starboard side will be reduced to 18% ${{=R}_{NWS}(SD}_{WS})=$ 0.18. On the other hand, performing the same manoeuvre at the ship’s speed reduced to half ahead (HAH-FAS) will result in generating the navigational risk factor of only 5% = ${{R}_{NWS}(SD}_{WS})=0.05$ (see Table 4).

## Conclusions

This paper presents the numeric indicators of navigational risk *R*_*N*_ as estimated for nine representative ship types with reference to navigational obstacles situated on the ship’s port side and starboard side respectively. However, a comprehensive analysis of the navigational risk in the navigable sea area analysed requires that the distribution of all navigational hazards situated within the three XYZ axes as a function of relevant parameters of the ship’s 3D domain model is studied.

The paper compares three types of domain parameters according to the guidelines by the PIANC, Coldwell and Rutkowski (3D). The results obtained for *R*_*N*_ indicators at times assume radically different values, whereby the domain model by Rutkowski seems to be the most accurate one. To sum up, according to the 2D domain method by Coldwell and the 3D domain method by Rutkowski, navigating the following ship types: fisher ship (D), high speed water jet rescue ship (E), fishing boat (F) and z-drive prevention response tug (I), proves to be completely safe. The analyses that were conducted required the use of appropriate hydrometeorological data for the area under consideration.

The presented method can be perceived as a universal one, as it depends only on the interrelation between the ship position and the position of the detected navigational obstacle, which obstacle may be land, another ship or object (e.g. an offshore installation), or a hydrometeorological factor generating a risk to the safety of navigation within a given navigable sea area (an open and/or restricted one).

## Data Availability

The data that support the findings of this study are to be provided by the first author Grzegorz Rutkowski (g.rutkowski@wn.umg.edu.pl) upon justified request.
